# Supplementary material for: Mapping of morpho-electric features to molecular identity of cortical inhibitory neurons
Source: PLoS Comput Biol. 2023 Jan 5;19(1):e1010058. doi: 10.1371/journal.pcbi.1010058 (PMC9815626; doi:10.1371/journal.pcbi.1010058)
Supplement: S1 Appendix — (DOCX) [file pcbi.1010058.s001.docx]

**S1 Appendix: electrophysiological features list:**

AHP_depth : relative voltage values at the first after-hyperpolarization (after the first spike)

AP1_amp : amplitude of the first action potential

AP2_amp : amplitude of the second action potential

AHP_time_from_peak : time between action potential peaks and first AHP depths

AP_amplitude : list of amplitudes of all the action potentials detected

AP_duration_half_width : width of spike at half spike amplitude

AP_begin_width : width of spike at spike start

mean_frequency : the mean frequency of the firing rate

burst_number : the number of bursts

voltage_base : membrane potential before stimulus (i.e. resting potential if no holding current is applied)

inv_time_to_first_spike : inverse time to first spike after application of stimulus (if a spike is detected)

inv_first_ISI : inverse of the first inter-spike interval (i.e. time between the first and second spike)

inv_second_ISI : inverse of the second inter-spike interval (i.e. time between the second and third spike)

inv_third_ISI : inverse of the third inter-spike interval

inv_fourth_ISI : inverse of the fourth inter-spike interval

inv_fifth_ISI : inverse of the fifth inter-spike interval

APlast_amp : amplitude of the last action potential detected

AP_begin_voltage : membrane potential at which the action potential starts

ISI_CV : The coefficient of variation of the inter-spike intervals

ISI_log_slope : The slope of a linear fit to a loglog plot of the inter-spike interval values

fast_AHP : Fast component of the after-hyperpolarization potential

adaptation_index2 : Normalized average difference of two consecutive inter-spike intervals

AHP_slow_time : time difference between absolute voltage values at the first after-hyperpolarization and peak, divided by interspike interval

doublet_ISI : Inter spike interval of doublet spikes (i.e. burst of two spikes)

decay_time_constant_after_stim : decay time constant measured from the end of the stimulus back to the voltage_base

mean_AP_amplitude : mean of the amplitudes of all the action potentials detected
