# Supplementary material for: Mapping of morpho-electric features to molecular identity of cortical inhibitory neurons
Source: PLoS Comput Biol. 2023 Jan 5;19(1):e1010058. doi: 10.1371/journal.pcbi.1010058 (PMC9815626; doi:10.1371/journal.pcbi.1010058)
Supplement: S2 Appendix — (DOCX) [file pcbi.1010058.s002.docx]

**S2 Appendix: NeuroM features list:**

total_length : Cumulated length of all neurites

total_length_per_neurite : Neurite lengths.

neurite_lengths : average neurite lenghts

terminal_path_lengths_per_neurite:

section_lengths : Section lengths.

section_term_lengths : Termination section lengths.

section_bif_lengths : Bifurcation section lengths

neurite_volumes : volume of neurites

neurite_volume_density : The volume density is defined as the ratio of the neurite volume and the volume of the neurite’s enclosung convex hull

section_volumes : volume of sections

section_areas : surface area of sections

section_tortuosity : tortuosities of sections

section_path_distances : Path lengths of sections

number_of_sections : Number of sections

number_of_sections_per_neurite : Number of sections for each neurites

number_of_neurites : number of neurites in the morphology

number_of_bifurcations : number of bifurcation points

number_of_forking_points : number of forking points

number_of_terminations : number of end points

section_branch_orders : Section branch orders

section_term_branch_orders : Termination section branch orders.

section_bif_branch_orders : Bifurcation section branch orders

section_radial_distances : Section radial distances

section_bif_radial_distances : Get the radial distances of the bf sections

section_term_radial_distances : Get the radial distances of the termination sections

section_end_distances : Section end to end distances

section_strahler_orders : Inter-segment opening angles in a section

local_bifurcation_angles : Get a list of local bifurcation angles

remote_bifurcation_angles : Get a list of remote bifurcation angles

partition : Partition at bifurcation points

partition_asymmetry : Partition asymmetry at bifurcation points

partition_pairs : Partition pairs at bifurcation points

partition_asymmetry_length : 'partition_asymmetry' feature with variant='length'

sibling_ratio : Sibling ratios at bifurcation points

diameter_power_relation : Calculate the diameter power relation at a bifurcation point

number_of_segments : Number of segments

segment_lengths : Lengths of the segments.

segment_areas : Surface areas of the segments

segment_volumes : Volumes of the segments

segment_radii : Arithmetic mean of the radii of the points in segments

segment_midpoints : List of segment mid-points

segment_taper_rates : Diameters taper rates of the segments

segment_path_lengths : Pathlengths between all non-root points and their root point

section_taper_rates : Diameter taper rates of the sections from root to tip

segment_radial_distances : list of distances between all segment mid points and origin

segment_meander_angles : Inter-segment opening angles in a section

principal_direction_extents : Principal direction extent of neurites the morphology

total_area_per_neurite : Total area of each neurites of the morphology
