# Supplementary material for: Mapping of morpho-electric features to molecular identity of cortical inhibitory neurons
Source: PLoS Comput Biol. 2023 Jan 5;19(1):e1010058. doi: 10.1371/journal.pcbi.1010058 (PMC9815626; doi:10.1371/journal.pcbi.1010058)
Supplement: S3 Appendix — (DOCX) [file pcbi.1010058.s003.docx]

**S3 Appendix: common me-features list:**

voltage_base

inv_time_to_first_spike

inv_first_ISI

inv_second_ISI

inv_third_ISI

inv_fourth_ISI

inv_fifth_ISI

decay_time_constant_after_stim

AHP_depth

AP1_amp

AP2_amp

AHP_time_from_peak

AP_amplitude

AP_duration_half_width

AP_begin_width

mean_frequency

burst_number

APlast_amp

AP_begin_voltage

ISI_CV

ISI_log_slope

fast_AHP

adaptation_index2

AHP_slow_time

doublet_ISI

mean_AP_amplitude

morpho_nm_total_length

morpho_nm_total_length_per_neurite

morpho_nm_section_lengths

morpho_nm_section_term_lengths

morpho_nm_section_bif_lengths

morpho_nm_section_volumes

morpho_nm_section_areas

morpho_nm_section_tortuosity

morpho_nm_section_path_distances

morpho_nm_number_of_sections

morpho_nm_number_of_sections_per_neurite

morpho_nm_number_of_neurites

morpho_nm_number_of_bifurcations

morpho_nm_number_of_forking_points

morpho_nm_section_branch_orders

morpho_nm_section_term_branch_orders

morpho_nm_section_bif_branch_orders

morpho_nm_section_radial_distances

morpho_nm_section_bif_radial_distances

morpho_nm_section_term_radial_distances

morpho_nm_section_end_distances

morpho_nm_section_strahler_orders

morpho_nm_local_bifurcation_angles

morpho_nm_remote_bifurcation_angles

morpho_nm_partition_pairs

morpho_nm_partition_asymmetry_length

morpho_nm_number_of_segments

morpho_nm_segment_lengths

morpho_nm_segment_areas

morpho_nm_segment_volumes

morpho_nm_segment_radii

morpho_nm_segment_midpoints

morpho_nm_segment_path_lengths

morpho_nm_section_taper_rates

morpho_nm_segment_radial_distances

morpho_nm_segment_meander_angles

morpho_nm_principal_direction_extents

morpho_nm_total_area_per_neurite

- 50 morphological moments
