# Supplementary material for: Mapping of morpho-electric features to molecular identity of cortical inhibitory neurons
Source: PLoS Comput Biol. 2023 Jan 5;19(1):e1010058. doi: 10.1371/journal.pcbi.1010058 (PMC9815626; doi:10.1371/journal.pcbi.1010058)
Supplement: S4 Appendix — (DOCX) [file pcbi.1010058.s004.docx]

**S4 Appendix: Neurite density moments extraction:**

Moments were computed using the same definition as in [1]:

$$m_{j,k}= \sum_{i=1}^{N} x_{i}^{j}y_{i}^{k}w_{i}$$

$\left( x_{i}, y_{i} \right)$ are the 2-D coordinates of the center of the ith segment and $w_{i}$ is the length of the arbor segment. Hence, the total length of the neurite shaft is given by the 0th order moment:

$m_{0}=m_{0,0}=\sum_{i=1}^{N} w_{i}$.

We modified the first equation to define a density moment within a layer $l$ as:

$$m_{j,k}^{(l)}=\sum_{i=1}^{N} x_{i}^{j}y_{i}^{k}w_{i} with {lim}^{(l-1)}<y_{i}<{lim}^{(l)}$$

We only considered moments for the special cases of j = 0, k > 0; and of j > 0, k =0 (separating x and y axis)

$m_{k}^{x, (l)}= \frac{m_{k,0}^{(l)}}{\sqrt{m_{0}}}$and

$$m_{k}^{y, (l)}= \frac{m_{0,k}^{(l)}}{\sqrt{m_{0}}}$$

with division by a necessary normalization in these cases [1]. We extracted $m_{0}$, $m_{k}^{x, (l)}$, $m_{k}^{y, (l)}$for $k \in[0, 2]$ and $l \in\left[ 1, 5 \right]$ for both BBP and AIBS morphologies. As stated above, $m_{0}$gives the total length of the neurite shaft whereas $m_{1}^{x, (l)}$ and $m_{2}^{x, (l)}$ relate to the mean and standard deviation of the neurite shaft density, respectively, along the *x* axis. Thus, for a given morphology we extracted 5 moments$\left( m_{0}^{(l)}, m_{1}^{x,(l)}, m_{2}^{x,(l)}, m_{1}^{y,(l)}, m_{2}^{y,(l)} \right)$ multiplied by 5 layers (L1, L2/3, L4, L5, L6) multiplied by 2 neurite types $\left( axon, dendrites \right)$resulting in 50 moments. If a neurite shaft did not extend to a given layer, all moments values were set at zero for that layer.

**Reference:**

1. Snider J, Pillai A, Stevens CF. A Universal Property of Axonal and Dendritic Arbors. Neuron. 2010 Apr 15;66(1):45–56.
