# Supplementary material for: Mapping of morpho-electric features to molecular identity of cortical inhibitory neurons
Source: PLoS Comput Biol. 2023 Jan 5;19(1):e1010058. doi: 10.1371/journal.pcbi.1010058 (PMC9815626; doi:10.1371/journal.pcbi.1010058)
Supplement: S5 Appendix — (DOCX) [file pcbi.1010058.s005.docx]

**S5 Appendix: Brief description of maker densities extraction:**

Briefly, marker expressions from *in-situ* hybridization experiments were aligned on a reference digital brain and converted to densities using reference values from literature. The reference brain was voxelized and densities of PV, SST and VIP expressing cells were extracted for each voxel across the whole brain. A density for inhibitory cells expressing none of the previously mentioned markers (REST) was also given by the Blue Brain cell atlas.
