# Supplementary material for: Mapping of morpho-electric features to molecular identity of cortical inhibitory neurons
Source: PLoS Comput Biol. 2023 Jan 5;19(1):e1010058. doi: 10.1371/journal.pcbi.1010058 (PMC9815626; doi:10.1371/journal.pcbi.1010058)
Supplement: S6 Appendix — (DOCX) [file pcbi.1010058.s006.docx]

**S6 Appendix: Formal definition of R-value.**

We implemented the R value as described in [1], using the same notation:

- *n* is the number of classes
- $C_{i}$the set of instances belonging to class $i$
- $U$the set of all instances $U= C_{1} \cup C_{2} \cup\ldots\cup C_{n}$
- $P_{i,m}$the m-th instance of class $i$
- $\lambda\left( x \right)= \left\{ \begin{matrix} 1, if x>0 \\ 0, otherwise \end{matrix} \right.$
- $kNN(P, S)$ the subset of *k*-nearest neighbors of instance *P* that belongs to the set of instance *S*
- $\theta$ threshold value above which the instance is considered as belonging to an overlapping region

The *R-value* for a class *i* is defined as:

$$R\left( C_{i} \right)= \frac{1}{\left| C_{i} \right|}\sum_{m=1}^{\left| C_{i} \right|} \lambda\left( \left| kNN\left( P_{i,m}, U- C_{i} \right) \right|- \theta\right)$$

Then, the *R-value* for a dataset f is defined as:

$$R\left( f \right)= \frac{1}{\left| U \right|}\sum_{i=1}^{n} \sum_{m=1}^{\left| C_{i} \right|} \lambda\left( \left| kNN\left( P_{i,m}, U- C_{i} \right) \right|- \theta\right)$$

In their paper, [1] adapted the *R-value* using two classes $C_{pos}$ and $C_{neg}$, such as $U= C_{pos} \cup C_{neg}$. They introduced the imbalance ratio $IR= \frac{\left| C_{neg} \right|}{\left| C_{pos} \right|}$ and proposed the *augmented R-value*:

$$R\left( f \right)= \frac{1}{IR+1}\left( IR\cdot R\left( C_{neg} \right)+ R\left( C_{pos} \right) \right)$$

We wanted to be able to use the metric in a case where $n>2$ (i.e. the dataset is not divided in only $C_{pos}$ and $C_{neg}$). We thus used the general definition of the R value:

$R\left( f \right)= \frac{1}{\left| U \right|}\sum_{i=1}^{n} \sum_{m=1}^{\left| C_{i} \right|} \lambda\left( \left| kNN\left( P_{i,m}, U- C_{i} \right) \right|- \theta\right)$, where we fixed the k value as $k=400$and set $\theta_{i}=int\left( \frac{k}{2} \cdot\frac{\left| U-C_{i} \right|}{\left| U \right|} \right)$. Thus, the threshold value is corrected to consider imbalance between classes and can be used for more than 2 classes.

**Reference:**

1. Borsos Z, Lemnaru C, Potolea R. Dealing with overlap and imbalance: a new metric and approach. Pattern Anal Appl. 2018 May 1;21(2):381–95.
