# Supplementary material for: Mapping of morpho-electric features to molecular identity of cortical inhibitory neurons
Source: PLoS Comput Biol. 2023 Jan 5;19(1):e1010058. doi: 10.1371/journal.pcbi.1010058 (PMC9815626; doi:10.1371/journal.pcbi.1010058)
Supplement: S1 Fig — We visualized all the pieces of information we had about each cell in a graph. Labels (dark and light blue), modality (e.g. morphological, electrical or genetic classification, dark red), morphological features (green), electrophysiological features (yellow), electrophysiological protocols (gold) were linked to individual cells from either Allen Institute for Brain Science (AIBS) or Blue Brain Project (dataset) for which this information was available. (PDF) [file pcbi.1010058.s008.pdf]

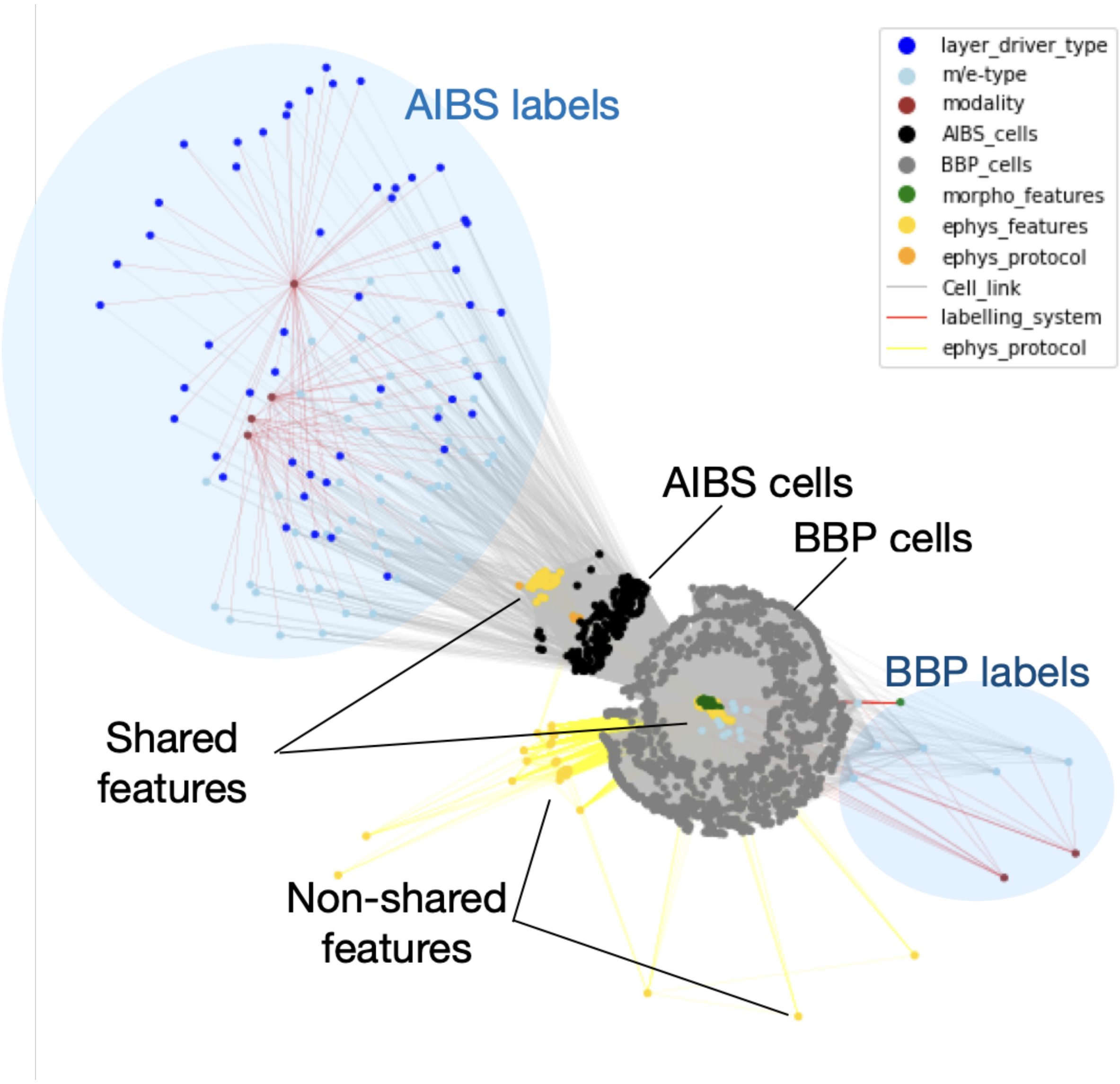

S1 Figure: Cell-centered "Knowledge Graph"

We visualized all the pieces of information we had about each cell in a graph. Labels (dark and light blue**)**, modality (e.g. morphological, electrical or genetic classification, dark red), morphological features (green), electrophysiological features (yellow), electrophysiological protocols (gold) were linked to individual cells from either Allen Institute for Brain Science (AIBS) or Blue Brain Project (dataset) for which this information was available.
