# Supplementary material for: Mapping of morpho-electric features to molecular identity of cortical inhibitory neurons
Source: PLoS Comput Biol. 2023 Jan 5;19(1):e1010058. doi: 10.1371/journal.pcbi.1010058 (PMC9815626; doi:10.1371/journal.pcbi.1010058)
Supplement: S2 Fig — Left—R-value computed for different values of α. Inset is a zoom of the dashed box. Right—Radius of the global dataset (AIBS +BBP) computed for different values of α. Radius computed as the mean of euclidean distance between each of the global dataset instances. Standard deviation from the euclidean distance between each of the instances are displayed in grey. (PDF) [file pcbi.1010058.s009.pdf]

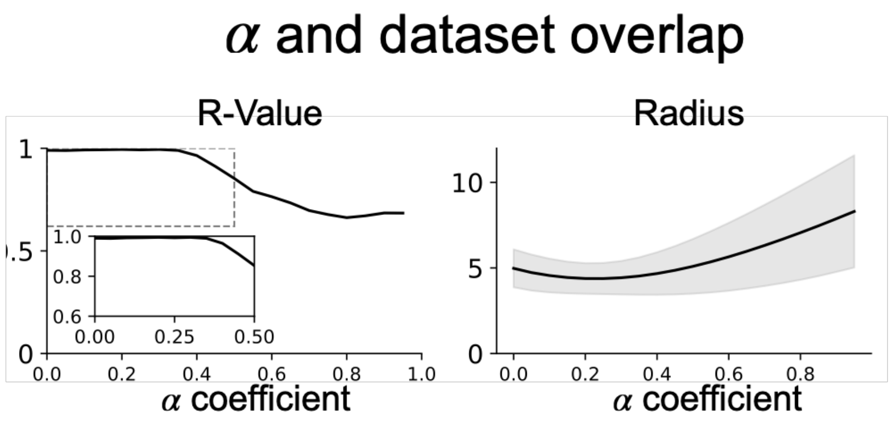

S2 Figure: Effect of alpha on datasets overlap (R-value). Left - R-value computed for different values of α. Inset is a zoom of the dashed box. Right - Radius of the global dataset (AIBS +BBP) computed for different values of α. Radius computed as the mean of euclidean distance between each of the global dataset instances. Standard deviation from the euclidean distance between each of the instances are displayed in grey.
