# Supplementary material for: Mapping of morpho-electric features to molecular identity of cortical inhibitory neurons
Source: PLoS Comput Biol. 2023 Jan 5;19(1):e1010058. doi: 10.1371/journal.pcbi.1010058 (PMC9815626; doi:10.1371/journal.pcbi.1010058)
Supplement: S3 Fig — Densities are computed as in Fig 5. Left—Profiles for somatosensory cortex (SSCtx). Right—Profiles for visual primary area (Visp). NGC: Neurogliaform cells; CHC: Chandelier cells; SBC: Small Basket cells; NBC: Nested Basket cells; LBC: Large Basket cells; MC: Martinotti cells; BTC: Bitufted cells; BP: Bipolar cells; DBC: Double Bouquet cells. (PDF) [file pcbi.1010058.s010.pdf]

Densities [mm^{-3}] along cortical depth

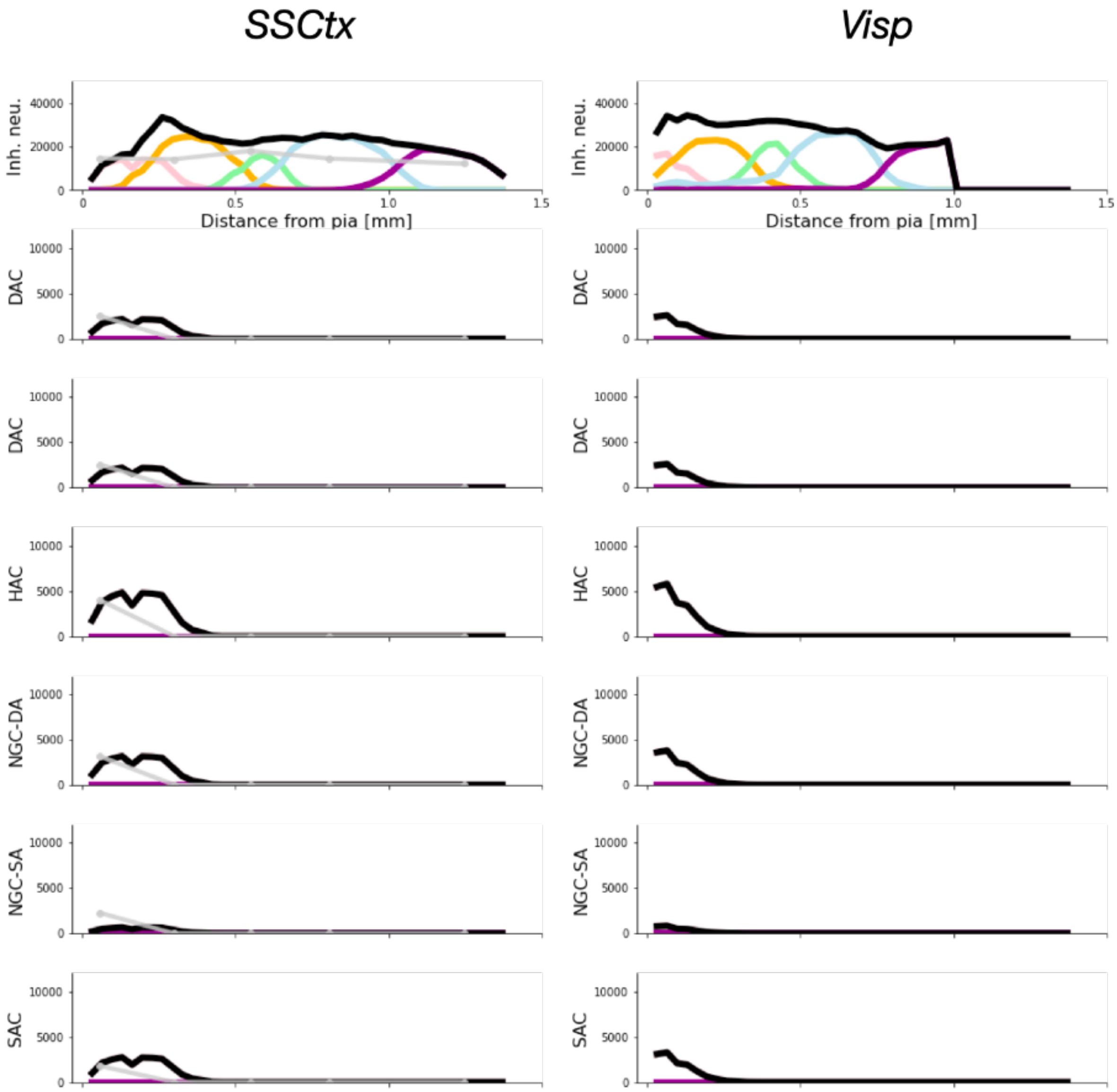

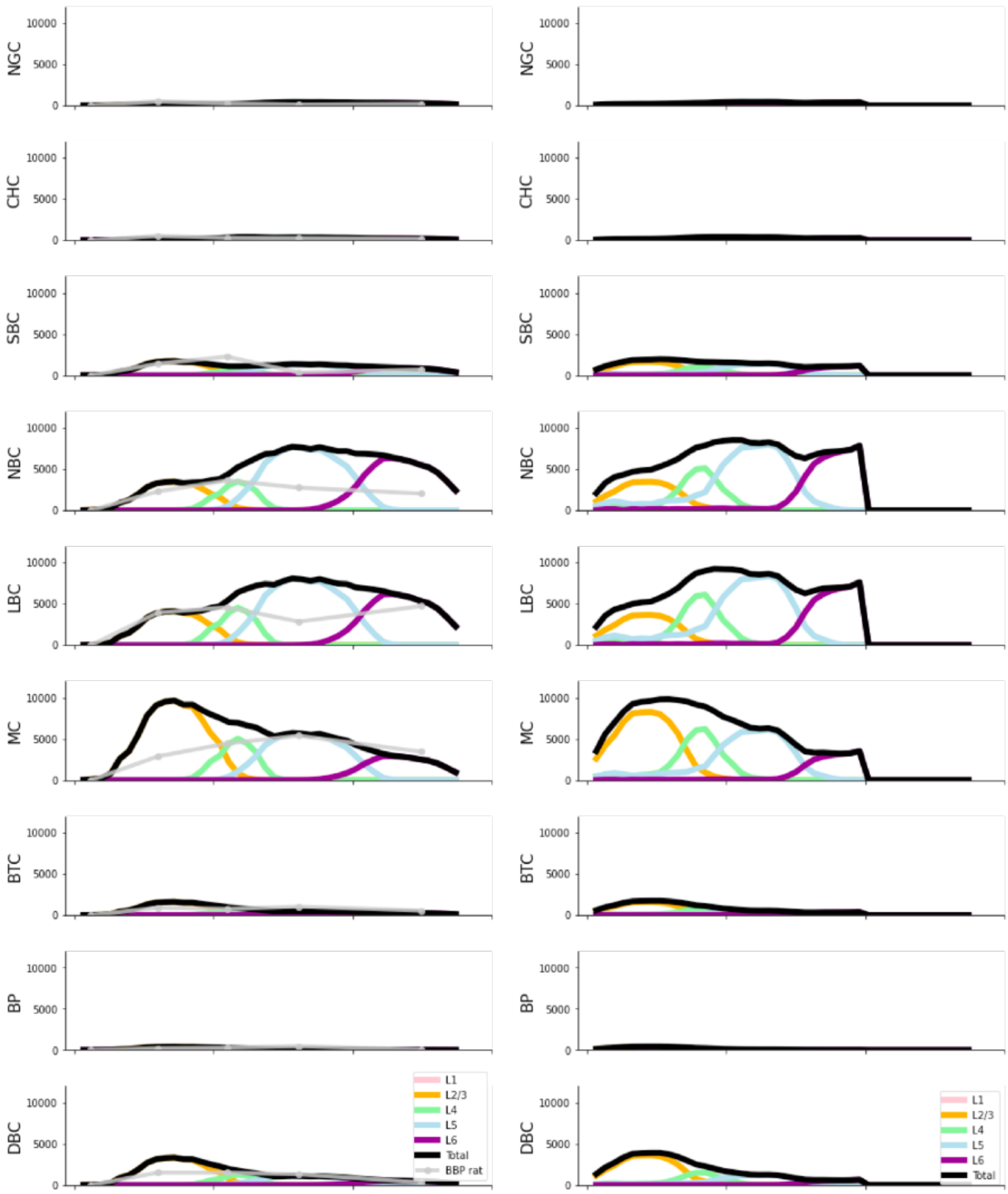

S3 Figure: Density profiles of BBP m-types computed from Blue Brain Mouse cell atlas densities combined with probabilistic mapping. Densities are computed as in Fig.5. Left - Profiles for somatosensory cortex (SSCtx). Right - Profiles for visual primary area (Visp). NGC: Neurogliaform cells; CHC: Chandelier cells; SBC: Small Basket cells; NBC: Nested Basket cells; LBC: Large Basket cells; MC: Martinotti cells; BTC: Bitufted cells; BP: Bipolar cells; DBC: Double Bouquet cells.
