# Supplementary material for: Mapping of morpho-electric features to molecular identity of cortical inhibitory neurons
Source: PLoS Comput Biol. 2023 Jan 5;19(1):e1010058. doi: 10.1371/journal.pcbi.1010058 (PMC9815626; doi:10.1371/journal.pcbi.1010058)
Supplement: S4 Fig — A. Mean m-type densities across cortical regions expressed in mm−3. The right column shows mean densities for the whole cortex. B. Divergence ratio from the mean density over the whole cortex for each m-type. Ratio was computed as rm−typeregion=meandensitym−typeregion−meandensitym−typeIsocortexmeandensitym−typeIsocortex. (PDF) [file pcbi.1010058.s011.pdf]

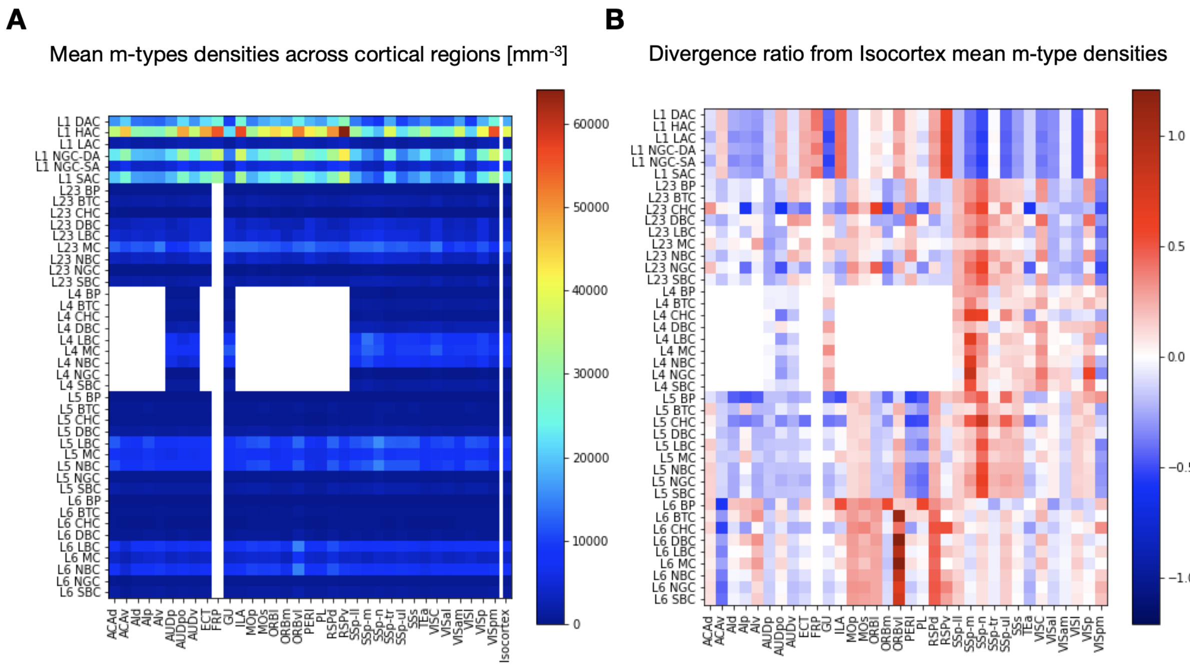

S4 Figure: A. Mean m-type densities across cortical regions expressed in mm^{-3}. The right column shows mean densities for the whole cortex. **B**. Divergence ratio from the mean density over the whole cortex for each m-type. Ratio was computed as $r_{m-type}^{region} = \frac{mean\ density_{m-type}^{region} - mean\ density_{m-type}^{Isocortex}}{mean\ density_{m-type}^{Isocortex}}$.
