# Supplementary material for: Mapping of morpho-electric features to molecular identity of cortical inhibitory neurons
Source: PLoS Comput Biol. 2023 Jan 5;19(1):e1010058. doi: 10.1371/journal.pcbi.1010058 (PMC9815626; doi:10.1371/journal.pcbi.1010058)
Supplement: S5 Fig — Cell counts were grouped by Cortical layer, common m-type (as defined in methods), native m-types, native e-types and molecular ID (when available). AIBS on the left and BBP on the right. Dashed lines represent the mean value for each case. The bottom right plot was created by applying the the probabilistic mapping P(marker|m-type) on m-type labels for the BBP dataset. It provides an estimate of the probable marker distribution in the BBP dataset, according to the mapping. (PDF) [file pcbi.1010058.s012.pdf]

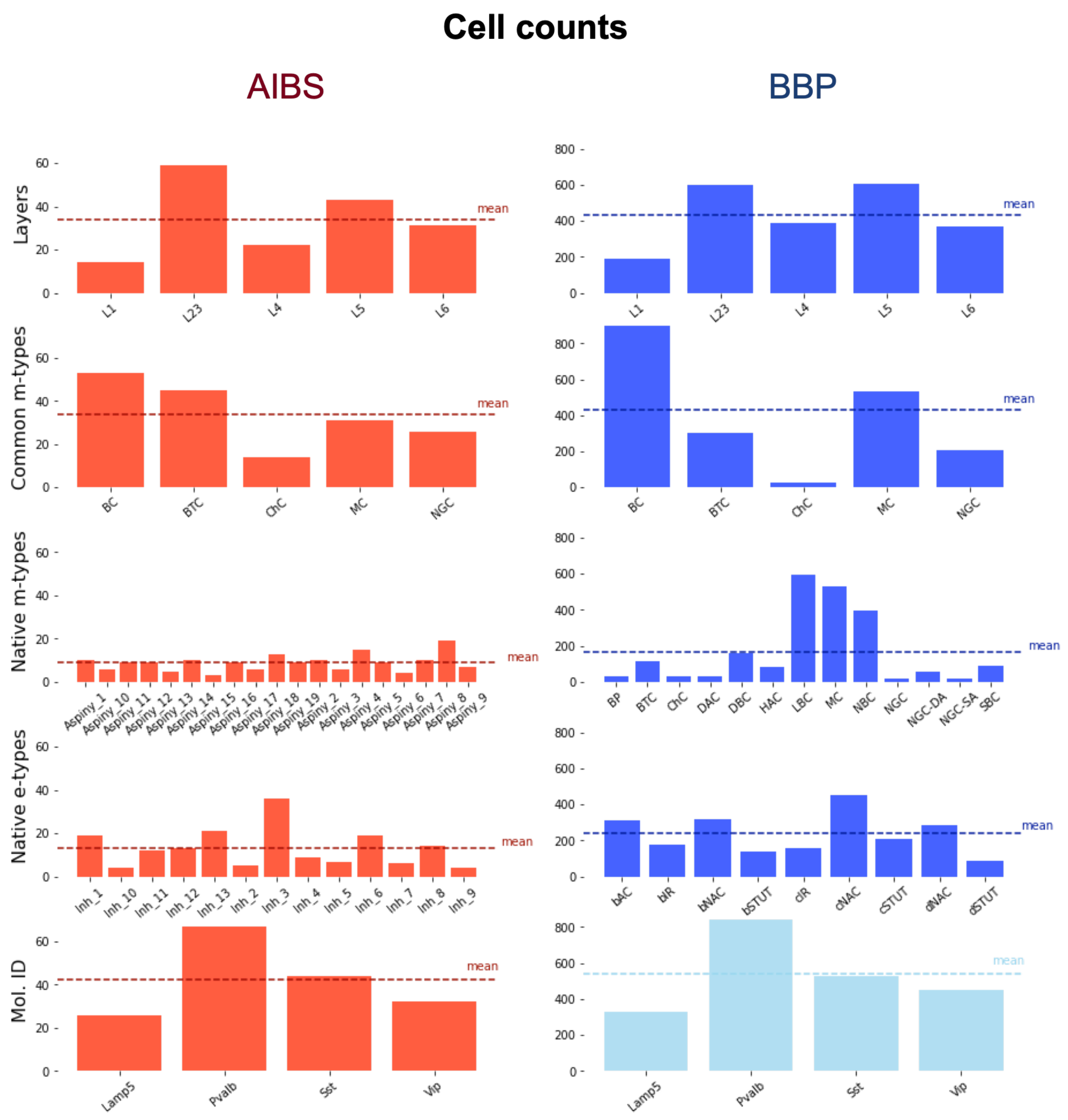

S5 Figure: Dataset cell composition according to different labelling systems. Cell counts were grouped by Cortical layer, common m-type (as defined in methods), native m-types, native e-types and molecular ID (when available). AIBS on the left and BBP on the right. Dashed lines represent the mean value for each case. The bottom right plot was created by applying the the probabilistic mapping P(marker|m-type) on m-type labels for the BBP dataset. It provides an estimate of the probable marker distribution in the BBP dataset, according to the mapping.
