# Supplementary material for: Mapping of morpho-electric features to molecular identity of cortical inhibitory neurons
Source: PLoS Comput Biol. 2023 Jan 5;19(1):e1010058. doi: 10.1371/journal.pcbi.1010058 (PMC9815626; doi:10.1371/journal.pcbi.1010058)
Supplement: S6 Fig — A. Mapping results when the pipeline was applied using the e-types general description provided by [13] for alpha optimization. AIBS neurons were assigned to one of the four following “common” e-types: Irregular spiking (IR), regular spiking (RS), fast spiking (FS) and adapting (Adapt.). Mapping was done between these “common” e-types and BBP e-types. B. Mapping results between molecular ID from AIBS dataset and BBP me-types with e-types labelled as either FS or nFS. Exactly the same morphologies are used to build me-models for both e-types. (PDF) [file pcbi.1010058.s013.pdf]

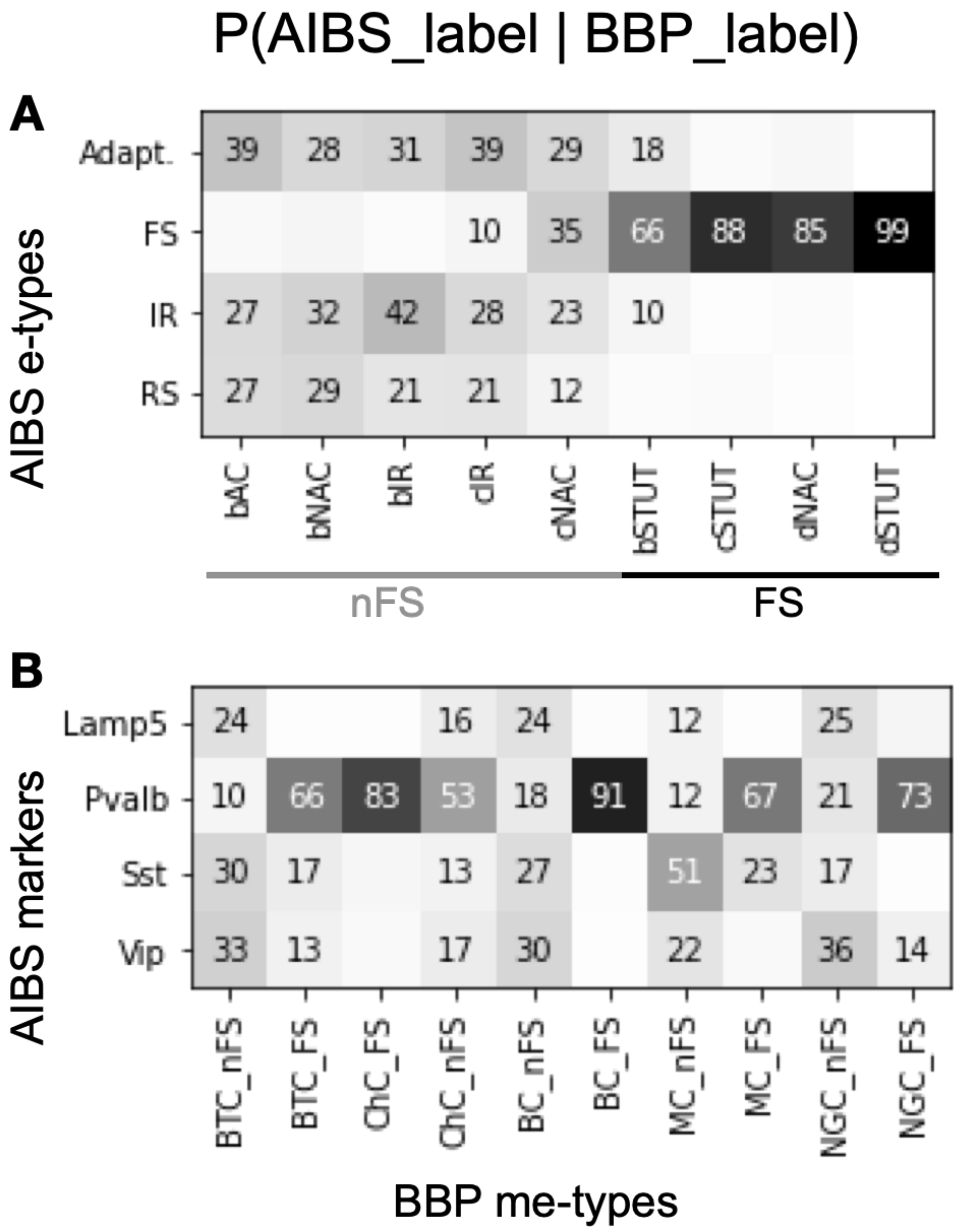

S6 Figure: E-types mapping and effect on molecular ID. A. Mapping results when the pipeline was applied using the e-types general description provided by [1] for alpha optimization. AIBS neurons were assigned to one of the four following "common" e-types: Irregular spiking (IR), regular spiking (RS), fast spiking (FS) and adapting (Adapt.). Mapping was done between these "common" e-types and BBP e-types. **B.** Mapping results between molecular ID from AIBS dataset and BBP me-types with e-types labelled as either FS or nFS. Exactly the same morphologies are used to build me-models for both e-types.

Reference:

1. Gouwens NW, Sorensen SA, Berg J, Lee C, Jarsky T, Ting J, et al. Classification of electrophysiological and morphological neuron types in the mouse visual cortex. Nat Neurosci. 2019 Jul;22(7):1182–95.
