# Supplementary material for: Mapping of morpho-electric features to molecular identity of cortical inhibitory neurons
Source: PLoS Comput Biol. 2023 Jan 5;19(1):e1010058. doi: 10.1371/journal.pcbi.1010058 (PMC9815626; doi:10.1371/journal.pcbi.1010058)
Supplement: S7 Fig — Projections for the e-features space (top), m-features space (middle) and me-features space (bottom) for the not normalized features, scaled morphologies and Z scaling cases. (PDF) [file pcbi.1010058.s014.pdf]

Principal components projected on features

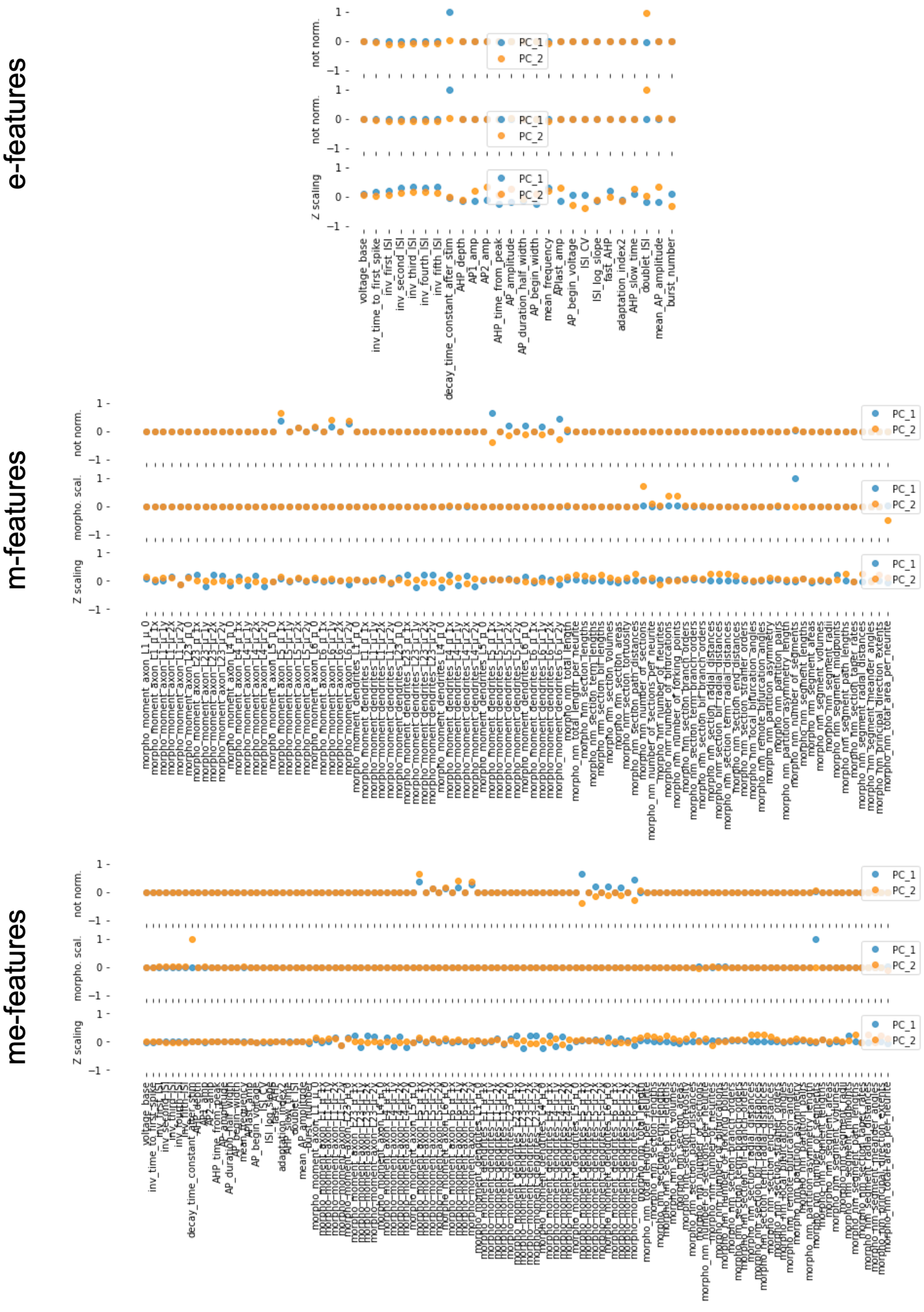

S7 Figure: Principal components from Fig. 2 projected on features. Projections for the e-features space (top), m-features space (middle) and me-features space (bottom) for the not normalized features, scaled morphologies and Z scaling cases.
