# Supplementary material for: Mapping of morpho-electric features to molecular identity of cortical inhibitory neurons
Source: PLoS Comput Biol. 2023 Jan 5;19(1):e1010058. doi: 10.1371/journal.pcbi.1010058 (PMC9815626; doi:10.1371/journal.pcbi.1010058)
Supplement: Attachment — Submitted filename: Review - Mapping of morpho-electric features to molecular identity of cortical inhibitory neurons.pdf [file pcbi.1010058.s015.pdf]

Mapping of morpho-electric features to molecular identity of cortical inhibitory neurons

The authors developed a method to assign a putative molecular identity to already available morpho-electrical (me-) rat neuronal models from the Blue Brain Project (BBP), using the information derived from a morpho-electrical and molecular dataset recorded in mice by the Allen Institute for Brain Science (AIBS).
Thanks to multiple normalization steps to 'align' the AIBS dataset to the BBP model, subsequent clustering according to a common me-space, and the probabilistic mapping between cells from the two datasets, the authors predicted the molecular identity of me-BBP modeled neurons.
Finally, the model prediction is thoroughly tested on a third database, as well as with the two already examined.
The paper focuses on cortical inhibitory neurons as their molecular identity is clearly established by the literature, contrary to excitatory neurons.

This contribution would add an important building block to our ability to model and understand the cortex, and even more it would constitute a valid attempt at bridging the gaps between the multiple databases available in the literature that span different species, brain areas, and types of data (e phys, morphology, and molecular).

The paper is written clearly, and the methodology used remarkable, and its few weak points examined openly in the discussion section.

However, I believe that the main hypothesis of this paper may not be so easy to take for granted and certainly might induce doubts in the audience and that should be discussed more clearly. This hypothesis is that two databases of neurons coming from different cortical areas, of different animal species, of different age groups (visual cortex of adult mice vs somatosensory cortex of young rats), are of straightforward comparison.

I would suggest the paper to be accepted, provided the authors can provide enough backing of this central hypothesis.

In more detail (**major points**)

> The density of specific cell types can vary depending on cortical layer, cortical area and age. An immediate example that comes to mind is the absence of a layer 4 in agranular cortex (e.g. PFC), though the differences present in the literature (some below) are several. The authors need to provide some support to their hypothesis that data from different species, age groups and cortical areas can be combined in their model in the first place.
>
> *Scala, Federico, et al. "Layer 4 of mouse neocortex differs in cell types and circuit organization between sensory areas." Nature communications 10.1 (2019): 1-12.*
>
> *Keller, Daniel, Csaba Erö, and Henry Markram. "Cell densities in the mouse brain: a systematic review." Frontiers in neuroanatomy 12 (2018): 83.*
>
> *Luebke, J. I., and Y-M. Chang. "Effects of aging on the electrophysiological properties of layer 5 pyramidal cells in the monkey prefrontal cortex." Neuroscience 150.3 (2007): 556- 562.*

Chang, Yu-Ming, et al. "Increased action potential firing rates of layer 2/3 pyramidal cells in the prefrontal cortex are significantly related to cognitive performance in aged monkeys." Cerebral Cortex 15.4 (2005): 409-418.

- A clear discussion about why such two databases can be used to complement each other is missing.

- For a similar reason, the clarity of the study would benefit from a description of the two databases, including specifying the neurons distribution according to animal age and sex (adding to what is present in S5 Figure regarding Layers and types).

- Only 15 layer 1 (L1) neurons are mentioned being taken from the AIBS database, would this number be a large and representative enough sample? The reference cited in the paper also claims that the existence of 4 interneuron subtypes in L1, making the sample of 15 rather slim.

  Schuman B, Machold RP, Hashikawa Y, Fuzik J, Fishell GJ, Rudy B. Four Unique Interneuron Populations Reside in Neocortical Layer 1. J Neurosci. 2019 Jan 2;39(1):125–39

  From Supplementary Figure S5, it is also clear how the sample is not uniformly distributed across layers or types.

  I am not convinced that the Layer 1 prediction should be presented with the same degree of certainty as the other predictions.

Minor Comments:

- Complementing Figure S5 even further with the predicted molecular ID of the BBP dataset, and with the third database used to test the model's performance on (Gouwens et al), would also be beneficial.
- Consider 'promoting' Figure S5 to a main figure to accompany Table 1 showing the actual predicted populations, or including the predicted molecular IDs in another main figure, as together with measures of accuracy, it would be interesting to have a clear representation of the actual prediction.
- The statement at line 69-71 might need a citation.
  "Additionally, neuronal classification based on molecular identity is not fully compatible with me-classification schemes. Nonetheless, many important results in the literature are formulated with respect to older schemes."
- Line 88: please specify which type (patch clamp?)
- Some of the equations submitted in the Supplementary material seem not to be displayed correctly (S6)
