# Supplementary material for: Mapping of morpho-electric features to molecular identity of cortical inhibitory neurons
Source: PLoS Comput Biol. 2023 Jan 5;19(1):e1010058. doi: 10.1371/journal.pcbi.1010058 (PMC9815626; doi:10.1371/journal.pcbi.1010058)
Supplement: Attachment — Submitted filename: Letter - answer to reviewers.pdf [file pcbi.1010058.s016.pdf]

Dear Drs Migliore and Marinazzo,

We wish to thank you for giving us the opportunity to submit a revised draft of our manuscript titled
"*Mapping of morpho-electric features to molecular identity of cortical inhibitory neurons*" to *PLOS Computational Biology*. We are grateful to you and all the reviewers for the constructive comments, which have allowed refinement of the manuscript content and analysis. In this document, we address the issues raised.

We provided more justification of the validity of the mouse and rat cell type comparison by extending the discussion with paragraphs supported by references from the literature. These paragraphs state that at low resolution levels (e.g. molecular classes such as PV+ or SST+) 1 - transcriptionally defined cell types are conserved between mouse and rat species and 2 - functional cell types have comparable features across species (e.g. PV+ cells are Fast spiking neurons and Martinotti cells project to L1). We also extended the principal component analysis to highlight which features were responsible for the mouse and rat cells heterogeneity or similarities across species, as suggested by reviewers 1 and 2. Finally, we incorporated the discussion points raised by reviewers about the use of the different cell-type labels and their impact on the alpha optimization process.

In more details:

Reviewer #1 comments:

- "*A discussion of what was learned from this extensive comparison about the similarities and differences between mouse and rat inhibitory neurons*" should be added.
  We further analyzed results from our PCA. We found that, in the electrophysiological features space, mouse cells scatter more along the decay-time constant while rat models scatter more along doublet inter-spike intervals. In the morphological space, axon and dendritic density moments are the most important component and the number of segments contributes as well but to a lesser extent. The latter is a source of bias (by the reconstructor) and is corrected by the Z-scoring. We added a discussion paragraph at lines 323-344 and included the principal components projection on features as supplementary figure S7.
- "*The notion that a normalization process can be used to convert a rat neuron into a mouse neuron or vis versa must be supported with a discussion of previous comparative studies*".
  We added a paragraph (lines 720-729) citing the work from Hodge et al. and Borobiak et al. that highlights the transcriptional similarities of cell-types across vertebrates as well as the work from Povysheva et al., Nigro et al. and Wang et al. to expose functional similarities.
- "*Were the datasets recorded with essentially the same solutions and temperatures?*"
  Recordings from both datasets happened in warm bath, 34 °C for AIBS (Gouwens et al 2019) and 35 °C for BBP (Le Bé et al. 2007). We now mention this point in the manuscript (lines 228-329).
- "*Additional recordings of rat neurons with single-cell genetics could validate [the approach]*"
  We agree, however we did not find rat data with single-cell genetic markers

combined with morphological reconstruction and electrophysiological recordings that could be exploited. We would be eager to improve our validation when this kind of data will become more readily available.

- "*What does alpha or the cross-dataset clustering suggest about the relative variability in morphology and electrophysiology within a molecularly defined cell type? This may be difficult to answer due to the different sizes of the datasets, but a discussion of the variability within cell types in rats and in mice may be useful.*"
  Thank you for the interesting suggestion. We added a discussion paragraph at lines 785-790 essentially stating that since the mapping process gets optimized results with a number of clusters much greater than the number of molecular markers, we should expect some variability in morphology and electrophysiology within a molecularly defined cell type. Otherwise, we would obtain an optimized mapping with a number of clusters around the number of molecular families. Other results from the literature already suggest that, even though molecular families are quite well defined regarding morphology and electrophysiology, large variability can still occur within a family (Gouwens et al., 2020; Scala et al., 2020).
- "*Further analysis of the 'species-specific' clusters could reveal new information about whether cell types differ across species.*"
  The point raised here is echoed by another comment from reviewer 2 suggesting that these clusters "could also be 'modeling-specific' clusters—combinations of e-features and models that are not found in the actual neuron population". We incorporated this point in the discussion about ''species-specific' clusters (lines 773-777) supported by an analysis of BBP me-type construction that assigned incompatible markers to m-types depending on the e-type attached to it (S6 Fig.).
- "*Fig2Aii and 2Aiii appear to be identical.*"
  Fig2Aii and 2Aiii appear identical because of the use of morphological features that have the largest variance (density moments) masking the variance from electrophysiological features. We confirm that electrophysiological features were indeed included in Fig. 2,A*iii* (as supported by S7 Fig., bottom panel).
- "*The link at the end of the results section doesn't work: http://cell-atlas.staging.nexus.ocp.bbp.epfl.ch/*"
  We apologize for the inconvenience, the official release of the portal has not been deployed yet. Meanwhile we can give access through an unofficial link: https://bbpteam.epfl.ch/project/user/rodarie/CA/

Reviewer #2 comments:
Major:

- "*It seems very odd that exactly 26 e-features would be selected by the knowledge graph analysis, and odder still that they have no indication of stimulus amplitude after all the discussion in the Methods about that approach.*"
  We concur, an important point was missing in the methods about the e-feature selection. To address this, we added more detailed explanations at lines 266-271. In essence, the 26 e-features from S1 were the targeted features to be extracted from different protocols. Thus we obtained for some of the targeted e-features values at different protocols. For example, we could have the action potential (AP) amplitude extracted at 100% of rheobase current but also at 150% and 200%. In this example, we would have three e-features related to AP amplitude linked to the cell in the knowledge graph, because of the different protocols. During feature selection, we

allowed a degree of freedom around the injected rheobase current. For example, if in neuron A, the AP amplitude was extracted at 120% of the rheobase but in neuron B it was extracted at 100%, we allowed the extraction for comparison as long as the difference in percentage was lower than 25%. Thus, we could obtain comparable values for all the 26 e-features presented in supporting information S1 extracted from very close protocols.

- "*How do the data sets differ in the first place, and how is z-scoring reconciling those differences? For example, Figure 2A is striking in that nearly all the variation in PC_1 is within AIBS cells and nearly all the variation in PC_2 is among the BBP models — what are the underlying e-features that are driving those differences? Similar, with the m-features, one of the most noticeable things in the non-normalized plot is that there appear to be a few extreme outliers among the BBP models driving the scale of the PCs, which go away after the layer-thickness normalization. How is the normalization changing those properties so dramatically?*"
  This comment is echoed by the first comment for reviewer 1.We addressed it above. The outliers arise from higher order moments and the size differences between rat and mouse neurons. Since the higher order moments follow square laws, a difference of 10 µm size of a neurite will result in a difference of 100 in these moments. This is why the largest morphologies from the rat appear as outliers. However, when the (x, y) coordinates are normalized relative to layer boundaries, the size differences will be eliminated.
- "*The authors state that finding consistent values of alpha for the AIBS data set but not the BBP data set is surprising; they would expect higher alpha values for m-only labels. They speculate that it may be due to the smaller size of the AIBS data set and/or an inability to predict the AIBS labels from the chosen features. However, could another factor be that the AIBS e-, m-, and me-labels are overall more congruent with each other than the BBP sets of labels?*"
  We agree with this analysis and incorporated these points in the discussion at lines 754-758. The congruence of the AIBS labels are supported by figure 5 c,d from Gouwens et al., 2019 paper that qualitatively show some correlation between e-, m-, and me-labels. It is also supported to a lesser extent by the fact that AIBS e-, m- and me-labels correlate well with marker labels.
- About Cross dataset validation: "*It isn't really explained why one direction (train on BBP, predict AIBS) works substantially better than the other direction.*"
  As reviewer 2 analyses in the previous point "*AIBS e-, m-, and me-labels [seem] overall more congruent with each other than the BBP sets of labels*" and develop later in the current point "*it seems like there is electrophysiological information available to be used [in all AIBS labels], which might be driving the alpha lower.*" This would lead to a decrease of the mapping efficiency in case of morphologies alone when training on the AIBS dataset. We fully agree with reviewer 2 analysis and incorporated these points into discussion at lines 824-830.
- "*Why were me-types used for the common labels instead of the AIBS m-types*"
  We had a description referring to types commonly used in literature for AIBS ME-types only. We now specify this at line 468. As for the grouping of ME_Inh_22, 23, and 26 we acknowledge that the choice could be surprising but we were limited by the consensus that had to be found between AIBS ME-types morphological description and BBP m-types description.

- “*I think the study would benefit from more careful investigation of how the inconsistencies manifest — what features are different, how does normalization and z-scoring affect those features, and where are the differences in the validation results arising from*”
  This is echoed by reviewer 2 's second comment and we have addressed it by extending the PCA analysis.
- “*I also feel as though the authors should be clearer that they are linking a set of partially randomized, simulated model data to a set of experimental recordings*”
  We have clarified this point by referring to the BBP model building process not only in the method section, as it was the case before, but also in the results (l. 520-522) and discussion sections (l. 714).
- "*I would be more excited to see this pipeline used to connect their large model database to an atlas of more finely defined transcriptomic types (perhaps based on merFISH or similar results) using something like a Patch-seq data set as the linker.* "
  We concur and would like to communicate that the refinement of the Mouse cell atlas to include merFISH and fine transcriptomic type data is currently under investigation at BBP. We added a sentence to promote the next iteration (l. 811-814).

Minor:

- “*The section starting on line 323 cites multiple parts of Fig 1D (i through iv) which don't exist. Is it intended to refer to Fig. 3A?*”
  It should refer to fig. 3D. We changed reference to fig.3D
- “*Fig 2 and the related text could be clearer that the ‘normalization’ refers to the layer-based morphological normalization, since z-scoring is also a form of normalization*”
  We changed ‘normalization’ to ‘morphological scaling’ in Fig. 2.
- “*At several points, the text states that excitatory neuron classes are equivalent to layer position; this oversimplifies the situation a bit too much, as it ignores projection type as the other major aspect of traditional excitatory neuron classification (e.g., pyramidal tract-projecting, intratelecephalic projecting, corticothalamic projecting)*”
  We agree and we added additional nuance and detail to the text (line 92-97 and lines 502-504).
- “*It is stated a few times that the authors use BBP m-types in a figure or discussion instead of the BBP me-types ‘for clarity’ - could they specify why it is clearer? Is it just because there are fewer m-types than me-types, or are there other reasons?*”
  Indeed, there are much fewer m-types (9) than me-types (>80). We added the clarification at line 545.
- “*The feature appendices (S1, S2, S3) also should have brief descriptions of the features so readers do not need to reference to separate documentation to decode the abbreviations.*”
  We added a brief description of features in the supplementary information.

Reviewer #3 comments:

Major:

- "*A clear discussion about why such two databases can be used to complement each other is missing.*"
  This comment echoes the second comment from reviewer 1. We addressed it above.
- "*For a similar reason, the clarity of the study would benefit from a description of the two databases, including specifying the neurons distribution according to animal age*

and sex (adding to what is present in S5 Figure regarding Layers and types)."
It would be interesting to know this distribution, however we could only retrieve the animals age and sex from Gouwens et al. (2019) but not for the BBP dataset. Therefore, we did not include these distributions.

- "*Only 15 layer 1 (L1) neurons are mentioned being taken from the AIBS database, would this number be a large and representative enough sample?*"
  We agree and specified that L1 neurons were used for completeness but results should be considered with caution. We added this clarification in the methods section - AIBS dataset description (lines 137-139) and in the results section (lines 575-578)

Minor:

- "*Complementing Figure S5 even further with the predicted molecular ID of the BBP dataset, and with the third database used to test the model's performance on (Gouwens et al), would also be beneficial.*"
  We agree with reviewer 3 that knowing the repartition of predicted labels in BBP dataset would be of interest. However, given that our approach provides probabilistic mapping, this analysis seems difficult to achieve. Still we estimated the distribution by dividing each me-types according to the probabilities of predicted markers and included it to figure S5 (while being clear about how we obtained this distribution).
- "*Consider 'promoting' Figure S5 to a main figure to accompany Table 1 showing the actual predicted populations, or including the predicted molecular IDs in another main figure, as together with measures of accuracy, it would be interesting to have a clear representation of the actual prediction.*"
  Thank you for the suggestion. We make it more clear now that the mapping is based on a probabilistic approach. We think it can be misleading to show the results stated above in a main figure as we really think that the pipeline results should be seen as a probabilistic mapping (shown in Fig. 5A). We thus kept the figure as supplementary information.
- "*The statement at line 69-71 might need a citation.'Additionally, neuronal classification based on molecular identity is not fully compatible with me-classification schemes. Nonetheless, many important results in the literature are formulated with respect to older schemes.*'"
  We added citations from Ascoli et al., 2008; DeFelipe et al., 2013; Emmenegger et al., 2018; Lim et al., 2018 at line 71.
- "*Line 88: please specify which type (patch clamp?)*"
  Recordings used are from patch-clamp experiments. We added "electrophysiological 'patch-clamp' recordings" at line 88.
- "*Some of the equations submitted in the Supplementary material seem not to be displayed correctly (S6)*"
  We apologize for this error, we made sure that the format was compatible for all equations in the document.

Reviewer #4 raised no major nor minor issue.

Once again, we thank you for the time you put in reviewing our paper and look forward to meeting your expectations. Since your inputs have been precious, in the eventuality of a publication, we would like to acknowledge your contribution explicitly.

The authors
